# Speciation in Western Scrub-Jays, Haldane’s rule, and genetic clines in secondary contact

**DOI:** 10.1186/1471-2148-14-135

**Published:** 2014-06-17

**Authors:** Fiona C Gowen, James M Maley, Carla Cicero, A Townsend Peterson, Brant C Faircloth, T Caleb Warr, John E McCormack

**Affiliations:** 1 Moore Laboratory of Zoology, Occidental College, Los Angeles, CA, USA; 2 Museum of Vertebrate Zoology, University of California, Berkeley, CA, USA; 3 Biodiversity Institute, University of Kansas, Lawrence, KS, USA; 4 Department of Ecology and Evolutionary Biology, University of California, Los Angeles, CA, USA; 5 Museum of Natural Science, Louisiana State University, Baton Rouge, LA, USA

**Keywords:** Birds, Speciation, Gene flow, Postzygotic reproductive isolation, Phylogeography

## Abstract

**Background:**

Haldane’s Rule, the tendency for the heterogametic sex to show reduced fertility in hybrid crosses, can obscure the signal of gene flow in mtDNA between species where females are heterogametic. Therefore, it is important when studying speciation and species limits in female-heterogametic species like birds to assess the signature of gene flow in the nuclear genome as well. We studied introgression of microsatellites and mtDNA across a secondary contact zone between coastal and interior lineages of Western Scrub-Jays (*Aphelocoma californica*) to test for a signature of Haldane’s Rule: a narrower cline of introgression in mtDNA compared to nuclear markers.

**Results:**

Our initial phylogeographic analysis revealed that there is only one major area of contact between coastal and interior lineages and identified five genetic clusters with strong spatial structuring: Pacific Slope, Interior US, Edwards Plateau (Texas), Northern Mexico, and Southern Mexico. Consistent with predictions from Haldane’s Rule, mtDNA showed a narrower cline than nuclear markers across a transect through the hybrid zone. This result is not being driven by female-biased dispersal because neutral diffusion analysis, which included estimates of sex-specific dispersal rates, also showed less diffusion of mtDNA. Lineage-specific plumage traits were associated with nuclear genetic profiles for individuals in the hybrid zone, indicating that these differences are under genetic control.

**Conclusions:**

This study adds to a growing list of studies that support predictions of Haldane’s Rule using cline analysis of multiple loci of differing inheritance modes, although alternate hypotheses like selection on different mtDNA types cannot be ruled out. That Haldane’s Rule appears to be operating in this system suggests a measure of reproductive isolation between the Pacific Slope and interior lineages. Based on a variety of evidence from the phenotype, ecology, and genetics, we recommend elevating three lineages to species level: *A. californica* (Pacific Slope); *A. woodhouseii* (Interior US plus Edwards Plateau plus Northern Mexico); *A. sumichrasti* (Southern Mexico). The distinctive Edwards Plateau population in Texas, which was monophyletic in mtDNA except for one individual, should be studied in greater detail given habitat threat.

## Background

The study of speciation has been influenced during the last decade by increasing awareness that different parts of the genome tell different evolutionary stories [1-3]. Previously, speciation studies relied heavily on mitochondrial DNA (mtDNA), given the numerous benefits offered by these data for inferring evolutionary history at diverse timescales (e.g., high substitution rates) [4]. Yet, along with these benefits come drawbacks [5], among them that mtDNA is a single recombinational unit that can present a biased snapshot of evolutionary history when mtDNA data are not balanced by independent evidence from nuclear markers.

One case in which mtDNA data can be particularly misleading occurs during gene flow between populations in secondary contact. Haldane was the first to note that when two differentiated lineages come back into reproductive contact, the heterogametic sex (i.e., the one with two types of sex chromosomes) often shows reduced fertility [6]. The mechanistic reasons behind Haldane’s Rule, as it came to be known, are under intense study [7-10]. While we may not understand why Haldane’s Rule occurs, the consequences for female-heterogametic species like birds and butterflies are clear: if female hybrids show reduced fertility, then mtDNA, which is inherited as a single recombinational unit from the female parent, is less likely to be passed on to the next generation. As a result, mtDNA might show little or no evidence of hybridization, despite the fact that hybridization occurs and paternal, nuclear alleles move between lineages.

The potential mismatch between gene flow estimated from mtDNA and gene flow estimated using nuclear markers is not just theoretical; it has been demonstrated empirically by studies of female-heterogametic organisms [11-18]. This phenomenon is of interest to biologists, among other reasons, because of the continuing influence of the Biological Species Concept (BSC) and its emphasis on cessation of gene flow as a necessary criterion for species delimitation [19]. While the BSC may not be applied as commonly in practice as is claimed [20], it is nevertheless evident that many attempts to split species taxonomically fail due to the existence of gene flow and the *perceived* importance of the BSC. As empirical evidence continues to mount demonstrating mito-nuclear discordance [21,22], the use of nuclear markers has become essential to species delimitation and the study of speciation.

**Figure 1:**
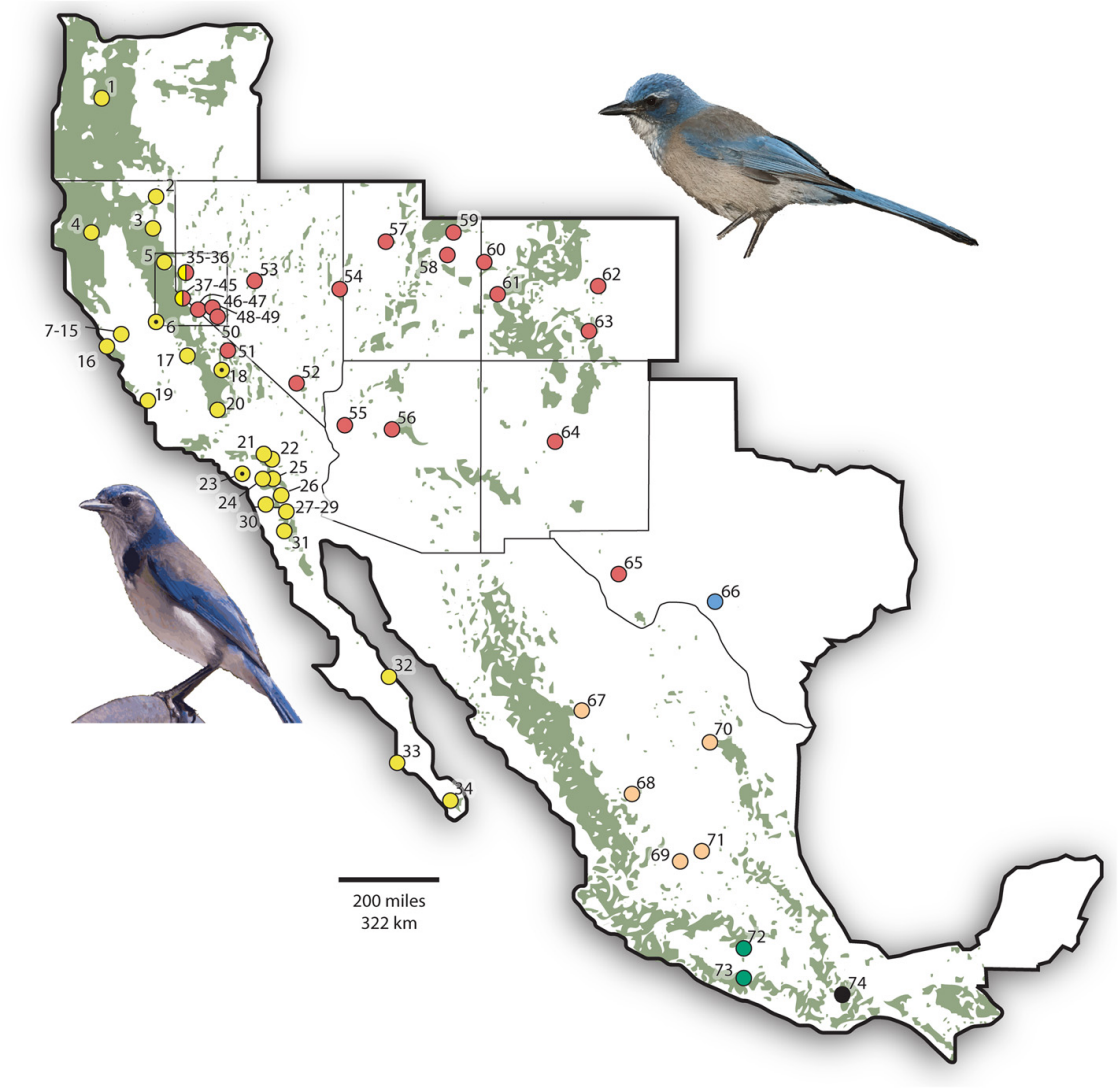
**Sampling locations for Western Scrub-Jays.** Green shading represents heavily forested regions that correspond with high-elevation barriers to dispersal. Numbers correspond to site numbers listed in Table 2. Dot color corresponds to the five nuclear genetic clusters from the Structure results shown in Figure 3. The square outline denotes the region of the hybrid zone depicted in greater detail in Figure 2. Dots with black points inside them represent the three coastal sites away from the hybrid zone that contain one or more interior mtDNA haplotypes. The scrub-jay on the lower left is representative of the coastal type, with bolder blue plumage, a larger blue collar, a bolder eyestripe, whiter undertail coverts, and a larger, more hooked bill compared to the interior type shown at upper right. Note that the range of the species extends into southwestern Canada and into some parts of the Great Plains that are not shown.

**Figure 2:**
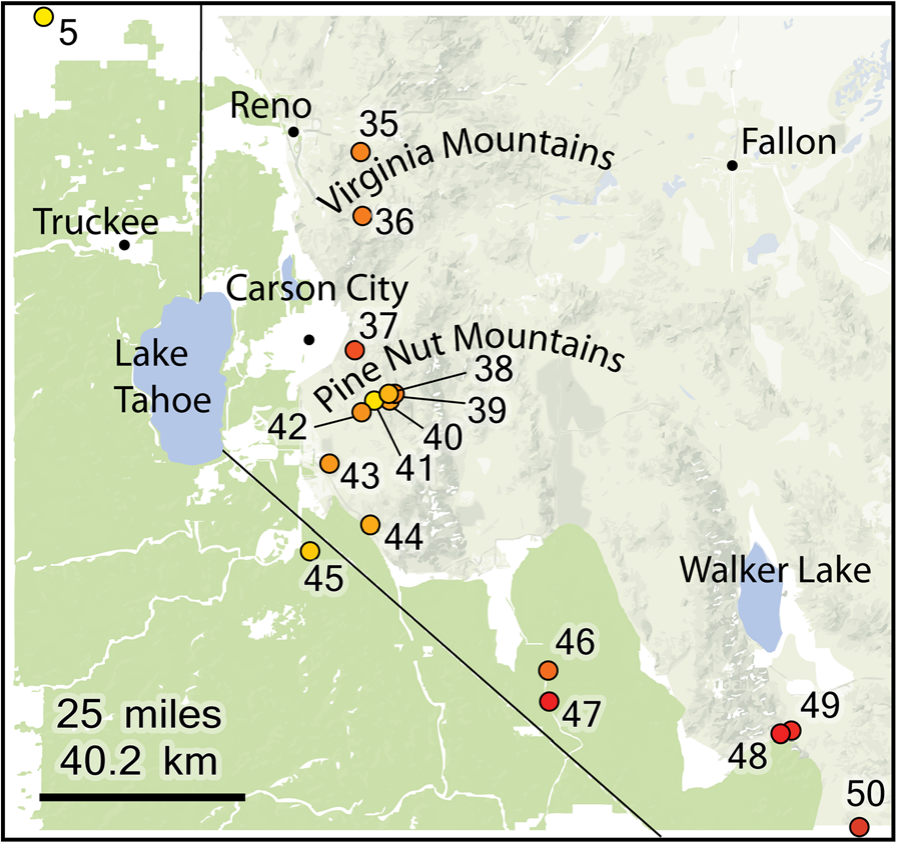
**Sampling sites in the hybrid zone.** Green shading corresponds to heavily forested regions considered to be a barrier to dispersal. Numbers correspond to site numbers listed in Table 2. The shading of the dots represent their cumulative Structure population assignment (including only individuals along the transect run at *K* = 2) averaged across all individuals in each population.

Western Scrub-Jays (*Aphelocoma californica*) are an excellent model for testing the mismatch between mtDNA and nuclear markers across a hybrid zone predicted by Haldane’s Rule because previous work documented a zone of secondary contact and hybridization between two divergent lineages. Western Scrub-Jays are widely distributed in North America from British Columbia, Canada to Oaxaca, Mexico and east to the Great Plains (Figure 1). Prior research delimited several lineages based on morphology, allozymes, and mtDNA variation [23-26]. Two of these lineages – one found primarily in oak woodlands along the Pacific slope and the other found in pinyon-juniper or pine-oak habitats in the interior US and Mexico – meet and hybridize in a few mountain ranges in western Nevada east of Lake Tahoe (Figure 2), but are otherwise separated from one another by the high-elevation conifer forests of the Sierra Nevada.

These two lineages (‘coastal’ and ‘interior’) are thought to have diverged from each other anywhere from 1 to 4 million years ago (Ma) based on divergence times calibrated with a fossil and estimated molecular substitution rates [24]. Coastal individuals are easily distinguished from those in the interior by their brighter blue plumage, bolder white supercilium, and more pronounced blue collar. Coastal individuals also have stout, hooked bills, which are better for husking acorns than the smaller, more pointed bills of interior individuals, which are better suited for extracting pine seeds from cones [27,28].

The hybrid zone was originally described by Pitelka [25] who traversed the zone and analyzed numerous museum specimens. He found intermingling of coastal and interior phenotypes in western Nevada, as well as individuals that appeared to be F1 hybrids. The hybrid zone has never been studied in detail using genetic techniques, although previous work documented a relatively rapid turnover of mtDNA types and mismatches of taxonomy and genotype of birds from this zone [23,24]. Nevertheless, a prior proposal to elevate the coastal and interior lineages to species status [29] failed because of doubts concerning the extent of gene flow in the contact zone.

The purpose of this study was to test for a prediction of Haldane’s Rule that there will be a mismatch between mtDNA and nuclear gene flow across the contact zone. Specifically, we predict that if Haldane’s Rule is operating in this system, we will see a narrower (i.e., steeper) cline in the transition of mtDNA haplotypes compared to nuclear markers. Before addressing this hypothesis, however, we needed to determine the exact nature of the contact zone in western Nevada and whether there were any undescribed contact zones nearby that might confound our results. We therefore first assessed broad-scale patterns of divergence and gene flow among Western Scrub-Jays across their entire geographic range, using deep sampling of populations and a suite of nuclear markers. Previous molecular studies sampled only one or a few individuals of different populations, focusing largely on allozymes [26] or mtDNA [23]. Our study design includes nearly 700 individuals sampled from throughout the range of the species, thus providing a comprehensive portrait of genetic divergence and allowing us to locate potentially undescribed contact zones. Finally, we correlated proportion of hybrid ancestry with phenotypic traits for a subset of individuals in the contact zone, as a way to control for the influence of environment and therefore test for a genetic basis of phenotypic differences between the coastal and interior lineages. If phenotypic traits are genetically based, we expect to see a correlation between neutral genetic assignment to coastal or interior lineages and the phenotypic differences known to characterize those lineages.

## Results

### Geographic structure of coastal and interior mtDNA types

To assess gene flow throughout the range of Western Scrub-Jays, we conducted simple mtDNA typing of all 689 individuals using restriction digest, which showed that most populations are composed entirely of coastal or interior mtDNA types, with very few populations showing mixing of mtDNA types. The four populations showing mixing were (1) the previously described contact zone in western Nevada, which featured several populations having mixed types 100 km east and southeast of Reno and Carson City (sites inside the inset square in Figure 1); (2) a single interior type (among n = 16 coastal types) found at the base of the western Sierra 100 km east of Stockton, CA (site 6); (3) an even mix of coastal and interior types in the eastern Sierra Nevada to the west of the Owens Valley near Independence, CA (site 18); and (4) one remarkable interior type (among n = 16 coastal types) in the Santa Ana Mountains in Orange County, Los Angeles (site 23).

### Broad-scale geographic structure of microsatellite variation

To complement the mtDNA typing results with information from nuclear markers, we genotyped all 689 individuals at 14 microsatellite loci. No pairs of loci were physically linked based on linkage tests. Of the 14 loci, one locus (MJG8) showed significant deviations from HWE across many populations following Bonferroni correction. We removed this locus from the data set. Four other loci showed deviations from HWE across some populations. We ran downstream analyses with and without these loci to investigate whether they were driving any of the patterns of genetic structure we observed. Results among analyses including and excluding loci were qualitatively similar, so we retained the larger group of 13 loci. No loci showed consistent signs of other microsatellite artifacts like large-allele drop-out or null alleles.

**Figure 3:**
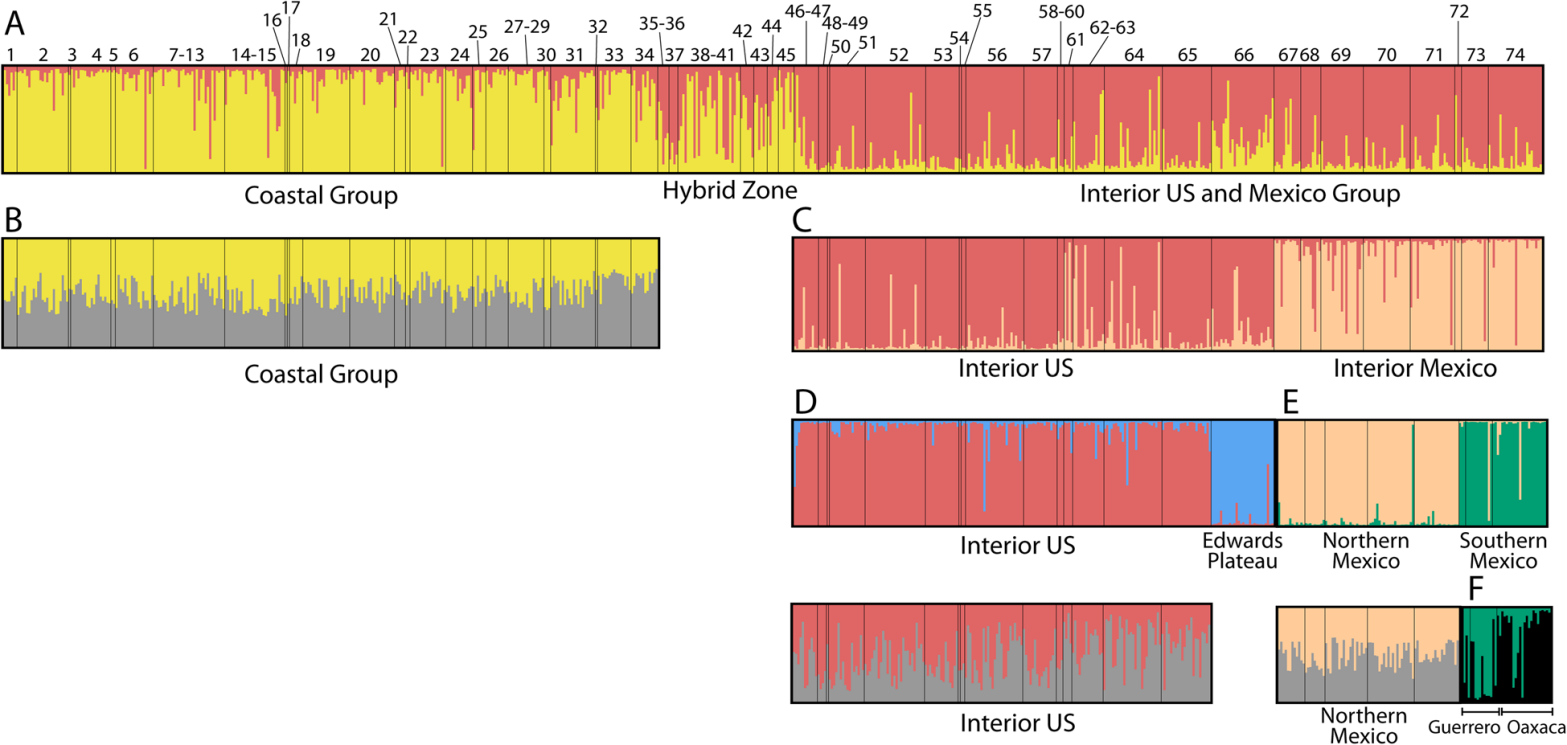
**Results from Structure runs on 13 nuclear microsatellite loci for successively smaller genetic clusters (*K*** **= 2 for each run).** Numbers correspond to sites listed in Table 2. **(A)** The first run split the individuals broadly into coastal and interior groups, with mixed assignment for individuals in the contact zone, which were removed from further runs. **(B)** The coastal group showed little geographic structure. **(C)** The interior group split into a US Interior cluster and a Mexico cluster. **(D)** An Edwards Plateau group split from the rest of US Interior; **(E)** A northern Mexico group split from a southern Mexico group; **(F)** The southern Mexico group showed some evidence for differential assignment between Oaxaca and Guerrero populations.

Initial Structure runs from *K* = 1–30 found increasing likelihood (LnL) values until *K* = 9, followed by a plateau in LnL until *K* = 13, followed by a decrease from *K* = 14–30. Structure runs on successively smaller clusters revealed five distinct geographic clusters of nuclear DNA variation (Figure 3) that were largely uniform in their population assignment. These genetic clusters were (1) a Pacific Slope group; (2) an Interior US group; (3) a group from the Edwards Plateau in Texas; (4) an interior group from Mexico north of the Transvolcanic Belt (Northern Mexico); and (5) an interior group from Mexico south of the Transvolcanic Belt (Southern Mexico).

### Phylogeny from mtDNA

**Figure 4:**
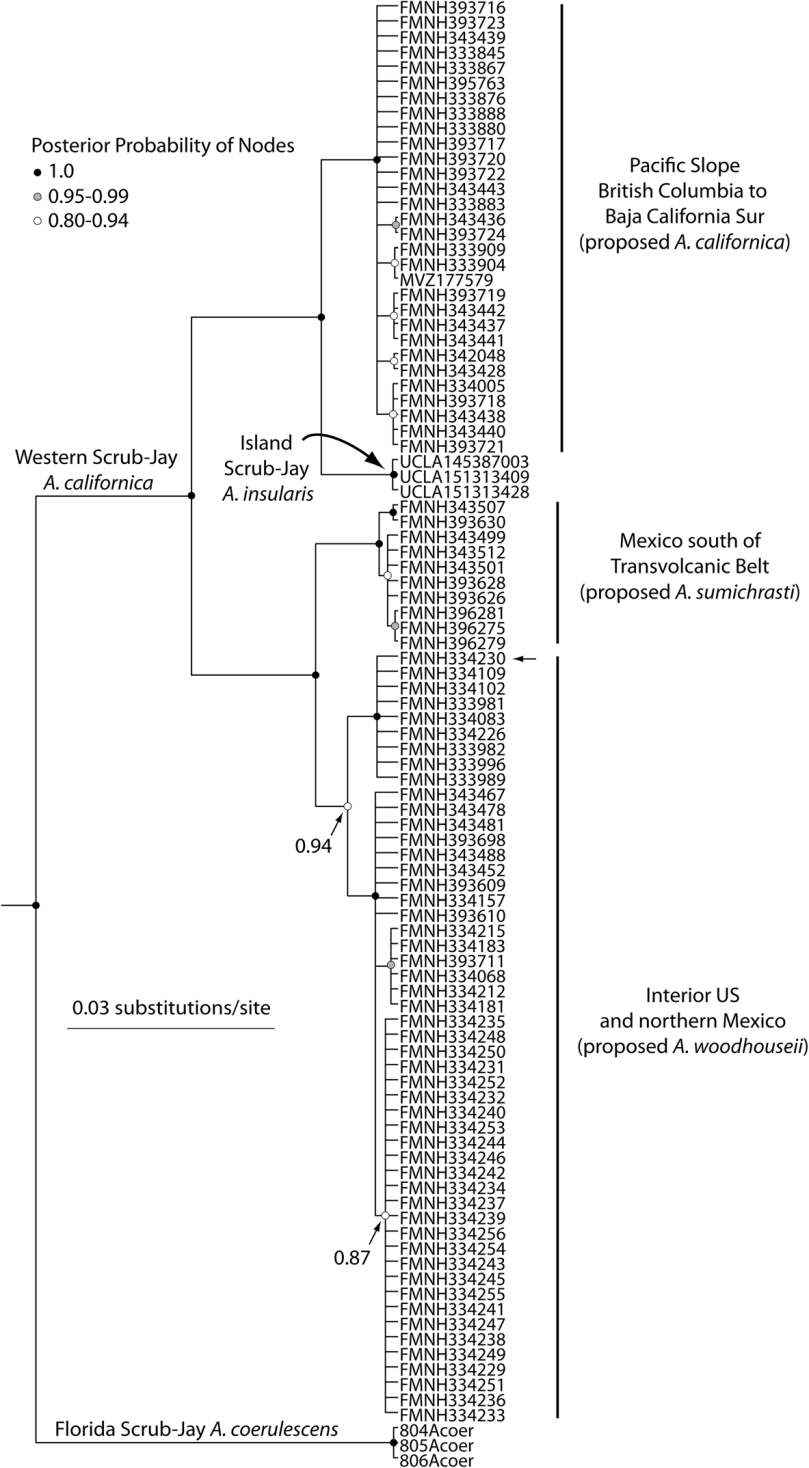
**BEAST consensus tree of scrub-jays based on *cyt b *mtDNA sequences new to this study combined with existing sequences from GenBank.** A more comprehensive version of this tree including all outgroup samples is available through Dryad [30]. The Western Scrub-Jay is paraphyletic because it does not include the Island Scrub-Jay. The one *texana* individual that does not group with the clade of *texana* individuals with 0.87 PP is denoted with a small arrow next to the tip label.

To determine if the novel genetic clusters found with microsatellites (e.g., the Edwards Plateau group) could also be found in a mtDNA phylogeny, we sequenced a subset of individuals at the *cytochrome b* gene (*cyt b*), including sampling from throughout the genus. We chose samples to both capture the major genetic lineages within Western Scrub-Jays and outgroups, and also to sample deeply within certain microsatellite groups of interest (e.g., we sequenced all individuals from the Edwards Plateau). The larger phylogeny, including all *Aphelocoma* samples, is available through Dryad [30]. In Figure 4, we show a trimmed version of this phylogeny including only scrub-jays, which confirms that the current concept of the Western Scrub-Jay is paraphyletic because it includes the Island Scrub-Jay (*A. insularis*). In addition to the Island Scrub-Jay, phylogenetic analyses placed three other highly supported and divergent clades within Western Scrub-Jays: (1) a Pacific Slope clade that was sister to the Island Scrub-Jay (1.0 posterior probability or PP); (2) a clade from Mexico south of the Transvolcanic Belt (Southern Mexico; 1.0 PP); (3) a clade of all other interior individuals (0.94 PP). Within this last clade, we recovered a weakly supported (0.87 PP) group from the Edwards Plateau in Texas, with the exception of one individual that grouped elsewhere in the interior clade (small arrow by terminal tip in Figure 4). There was no support in mtDNA for a split between Interior US and Northern Mexico (as was found in microsatellites), although some geographic localities grouped together with high PP.

### Geographic cline analysis

**Figure 5:**
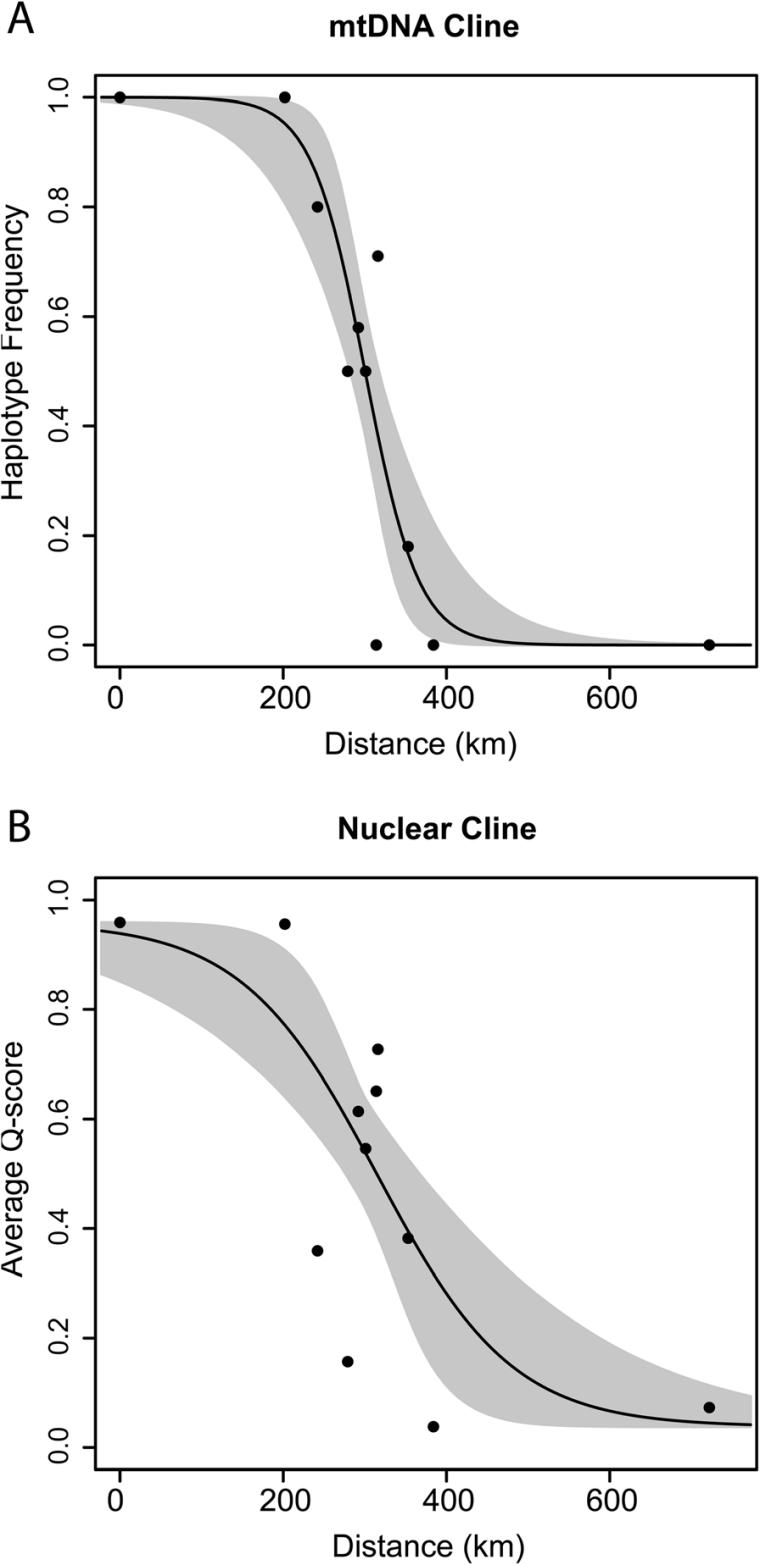
**Transition in genetic types across the hybrid zone for (A) mtDNA and (B) nuclear markers.** Black circles represent sampling localities, the dark line is the cline estimate and the gray shading represents confidence around the cline estimate.

Both the mtDNA and microsatellite data showed a step cline pattern of variation across the hybrid zone, and the null model of no clinal variation had much higher AICc scores in each case (mtDNA: null model = 96.11; cline model = 13.76; nuclear DNA: null model = 63.53; cline model = 15.72). Model 1 with no scaling was accepted as the most likely model for both datasets. Estimates of the center of the cline (*c*) were similar and had overlapping confidence intervals. The center of the mtDNA cline was ~300 km (280 – 319), and the center of the nuclear DNA cline was ~315 km (271 – 377). The width of the mtDNA cline was ~131 km (76 – 270), and the width of the nuclear DNA cline was ~331 km (145 – 678). The estimated width of the mtDNA cline fell outside the 2 log likelihood confidence intervals for the nuclear DNA, and vice versa, therefore the mtDNA cline was significantly narrower than the microsatellite cline (Figure 5). Disparity in dispersal distances between males and females did not account for the difference in cline width between mtDNA and microsatellites because our time-of-contact estimates under a model of neutral diffusion, which included estimates of male and female dispersal distance (females: σ = 1.5 – 5.0 km; males: σ = 0.5 – 3.0 km; [31]), did not overlap (mtDNA = ~690 – 7600 years, microsatellites = ~12,000 – 440,000 years). In other words, even taking sex-biased dispersal into account, mtDNA showed less diffusion.

### Correlation between phenotype and genotype

We observed strong correlations between hybrid values (Q-scores) from Structure and many phenotypic traits for the full data set of 123 individuals that included many pure coastal and interior individuals outside the hybrid zone, especially for plumage traits and principal components (PC) axes describing bill shape (Table 1; see Additional file 1 for PCA loadings table). However, by including individuals from allopatric ranges, this test potentially confounds genetic and environmental causes of trait variation. Within the hybrid transect and inside the core hybrid zone, where individuals experience the same environment, plumage traits, but not morphological traits, were correlated with Q-scores (Table 1).

**Table 1:** **Regression (*R***^**2**^**) values between hybrid index and phenotypic traits within and outside the hybrid zone**

**Trait**	**All**	**Transect**	**Core hybrid**
Wing	0.04*	ns	ns
Tail	ns	ns	ns
Tarsus	ns	ns	ns
Bill length	ns	ns	ns
Bill depth	ns	ns	ns
Bill width	0.11***	ns	ns
Morph PC1^1^	ns	ns	ns
Morph PC2^2^	0.06**	ns	ns
Morph PC3^3^	0.05**	ns	ns
Collar	0.38***	0.23**	ns
Vent	0.51***	0.46***	0.36***
Eyestripe	0.31***	0.25***	0.37***

^1^PC1 describes general size.

^2^PC2 describes a trade-off between body and bill size.

^3^PC3 describes a trade-off between bill length and bill width.

ns = not significant, *p < 0.05, **p < 0.01, ***p < 0.001.

## Discussion

### Haldane’s Rule and the mismatch in cline width between mtDNA and microsatellites

Our results support a key prediction from Haldane’s Rule that mtDNA will show a steeper cline across a hybrid zone than nuclear markers. Although Haldane’s Rule may explain the clinal pattern we observed, this pattern could also result from divergent selection on mtDNA types [32] or sex-biased dispersal, wherein females (and the mitochondrial genomes they pass on) tend to disperse less than males. Although we have not looked at selection, what little is known about natal dispersal in Western Scrub-Jays suggests that, to the contrary, females disperse about three times farther than males on average [31]. The results from our time-since-contact analyses, which incorporated estimates of dispersal distances, also support predictions from Haldane’s Rule, with less diffusion of mtDNA compared to nuclear markers.

Our results showing steeper clines in mtDNA compared to nuclear markers add to a gathering corpus of research showing discordant rates of DNA transmission across hybrid zones among different marker types [11-18,22], a pattern that has often been attributed to Haldane’s Rule [21]. Whatever the root cause, these results caution that conclusions about individual-level gene flow made on the basis of mtDNA alone may be underestimated. In our study, gene flow is generally restricted in both mtDNA and nuclear markers, due to the low dispersal distances of Western Scrub-Jays. In other species, such as long-distance dispersers with no sex-biased dispersal, mismatches could be much more pronounced.

Recent work on the genetic basis for Haldane’s Rule suggests it results from incompatibilities among genes that are exposed to selection on hybrid female Z chromosomes, causing sterility [7], whereas only those incompatibilities that are dominant are visible to selection in hybrid ZZ males. If Haldane’s Rule is indeed the cause of the clinal mismatch we observed, then our results suggest that the coastal and interior lineages of Western Scrub-Jay have begun the process of reproductive isolation. Ultimately, Haldane’s Rule as the cause of the mismatch in marker introgression rates could be determined through field studies or captive mating experiments that document the reproductive viability of female offspring resulting from hybrid crosses [33].

### Clarifying the hybrid zone between coastal and interior Western Scrub-Jays

Our results also help clarify the nature of the hybrid zone between coastal and interior lineages of Western Scrub-Jays. The fact that our hybrid transect populations could be fit to a cline suggests that Pitelka [25] was correct that the major axis of gene flow is from the northwest to southeast, as coastal populations continue around the north side of the Sierra Nevada and meet with interior populations in western Nevada. However, the coastal influence of the Woodford site (45 in Figure 2) warrants further study. In addition to gene flow from the north, some gene flow may link the southern end of the Sierra Nevada to the Woodford site, with coastal individuals inhabiting canyons of the southeastern Sierra that were not sampled for this study.

Our results also support Pitelka’s [25] conclusions that the hybrid zone is narrow and the result of secondary contact. Even the oldest time estimates from our time-since-contact analysis across the hybrid zone (~437,000 years) are younger than divergence estimates of the original split between coastal and interior lineages [1 to 4 Ma; 24], supporting the hypothesis that the hybrid zone results from secondary contact and not *in situ* divergence. In terms of broader biogeographic evidence, several other species have secondary contact zones where coastal and interior lineages meet in this general region of the Great Basin [34,35].

Our results also confirm that the contact zone in western Nevada is the only place where coastal and interior lineages come into contact to any great degree. Outside of western Nevada, two sites (6 and 23 in Figure 1) appear to contain examples of rare, long-distance dispersers from pure interior populations because the nuclear DNA profile of the two individuals matches their mtDNA type (the interior mtDNA individual at site 6 was 97% interior in nuclear DNA, and the interior mtDNA individual at site 23 was 95% interior in nuclear DNA). In contrast, at a third site in the eastern Sierra Nevada (18 in Figure 1), the three individuals with interior mtDNA had strongly coastal nuclear DNA profiles (91%, 95%, and 89% coastal), suggesting that they were advanced backcrosses from older hybridization events (i.e., nuclear DNA profiles of F1s would be closer to 50/50). Thus, although there appears to be one major contact zone, the exceptions show that the reality of dispersal and gene flow among jays in and around the Sierras – and across the Mojave Desert farther south – is more complex. Fine-scale sampling, combined with habitat-based methods of identifying dispersal corridors [36], could provide a more complete picture of gene flow across and around the Sierra Nevada for this and other species.

Previous studies documented phenotypic differences between coastal and interior Western Scrub-Jays [25,28], but ours is the first to provide evidence for a genetic basis for some traits. There was a correlation between nuclear genetic variation and phenotypic traits, such as bill shape and plumage traits, when considering all individuals, even those from pure populations (Table 1). However, including individuals from different geographic locations potentially confounds environmental and genetic sources of phenotypic variation. Still, many of these relationships remained, for the plumage traits at least, within the contact zone where there are hybrid individuals and all variety of backcrosses. The correlation between proportion of interior vs. coastal genetic ancestry and phenotypic traits for individuals that were fledged in the hybrid zone and therefore experienced the same environment since fledging provides evidence for a genetic basis of plumage traits, at the very least. Why we did not observe similar relationships for bill traits is not clear, but could be due to low statistical power, stabilizing selection, or greater plasticity in bill traits. Further studies with more individuals and better genomic coverage will help discriminate between these alternate hypotheses, as will studies that attempt to identify the genetic loci or environmental pressures responsible for the phenotypic differences.

### Genetic differentiation across North America’s scrub woodland

Previous genetic studies of species inhabiting North America’s pine-oak and scrub woodland have uncovered unexpectedly deep phylogenetic structure within species [37,38]. Results of the current study, which are the most comprehensive in terms of geographic, individual, and genetic sampling of Western Scrub-Jays, indicate at least five genetic clusters with strong spatial structuring (colored dots in Figure 1). The Pacific Coast and Southern Mexico groups have been recovered previously in studies of phenotype, allozymes, and mtDNA [23-26]. The Edwards Plateau group and the split between Interior US and Northern Mexico individuals are novel, although the inferred population clusters align with current subspecies designations [25].

This study is the first to show clear evidence of nuclear and mtDNA differentiation of Western Scrub-Jays on the Edwards Plateau in Texas (currently a separate subspecies, *A. c. texana*). All individuals in the Edwards Plateau population except one possess a unique mtDNA haplotype differing from other interior US haplotypes by two substitutions. Nuclear and mtDNA differentiation on the Edwards Plateau is interesting given the history of isolation of this region and its unique flora and fauna. The oak savanna and Ashe juniper woodland of the Edwards Plateau are separated from other scrub woodland to the west by an extension of the Chihuahuan Desert in the Pecos Valley. Fossil and pollen data suggest, however, that pygmy woodland was continuous across this region, and connected the Edwards Plateau to western forests throughout the last glacial maximum until about 11,000 years ago [39]. At least one other distinct bird species with a similar geographic range occurs on the Edwards Plateau, the Golden-cheeked Warbler (*Setophaga chrysoparia*), and other organisms in this region have distinct populations or subspecies [40,41]. Our results lend additional support to the distinctiveness of the biota in this region, where native habitat is under persistent conservation threat from urbanization.

The nuclear genetic break between the Interior US and Northern Mexico populations, occurring roughly where the Sierra Madre Occidental and Oriental turn into a network of sky islands near the US border, could be explained either by a break in suitable habitat or a sampling artifact. Addressing the latter point, observations posted on eBird (ebird.org) confirm observations of Western Scrub-Jays in southeastern Arizona and northern Sonora (in the vicinity of the genetic break), but these records could represent wandering individuals. Likewise few observations exist in the vicinity of the genetic break in southwestern Texas, and virtually no observations exist from northern Coahuila, where congeneric Mexican Jays (*Aphelocoma wollweberi*) occur at lower elevations, seemingly occupying the Western Scrub-Jay niche [25,42-44]. Thus, while available evidence suggests that the genetic break between Interior US and Mexico populations relates to a real distributional break near the US border, the exact causes of this break remain unclear. Future niche modeling could be used to determine if there are cryptic breaks in suitable habitat.

### Lack of genetic differentiation along the Pacific Coast

In contrast to the highly structured genetic portrait of the interior US and Mexico, Western Scrub-Jays along the Pacific Slope show a remarkable lack of genetic structure despite 2,500 km of nearly unbroken sampling from northern Oregon to the southern tip of Baja California. Lack of genetic structure over large distances suggests two possible scenarios: (1) high levels of dispersal and gene flow link coastal populations; or (2) recently expanded coastal populations have had insufficient time to accumulate genetic differences. Available evidence suggests that Western Scrub-Jays generally have low dispersal [31], notwithstanding a few rare, long-distance dispersal events discussed above. Furthermore, the original split between coastal and interior lineages occurred between 1–4 million years ago [24], providing ample time for genetic structure to emerge in coastal populations, absent other demographic events. Thus, the hypothesis that seems most likely is a recent expansion following a population bottleneck. However, Pleistocene niche models of Western Scrub-Jay niches suggest ample habitat along the Pacific Slope during the last glacial maximum [45]. Further study is needed to determine the cause of the apparent genetic homogeneity of Pacific Slope populations.

### Taxonomic recommendations

We recommend splitting the current concept of the Western Scrub-Jay into three species, as has already been suggested previously [46]. The Pacific Slope lineage (*A. californica*, including subspecies *californica, oocleptica, caurina, obscura, hypoleuca, superciliosa, immanis,* and *cactophila*) is phenotypically distinct [25], possesses a bill morphology adapted to local resources [27,28], is monophyletic in mtDNA, and is sister to another recognized species, the Island Scrub-Jay [23,24]. This study shows that the Pacific Slope lineage is also well-differentiated in nuclear markers and has only a narrow zone of contact with interior populations. The genetic clines across the hybrid zone are generally steep, and the mismatch between mtDNA and nuclear clines suggests a measure of reproductive isolation. These results would appear to meet criteria for species distinction under even the most stringent species concept.

Southern Mexico populations (*A. sumichrasti*, including subspecies *sumichrasti* and *remota*) also meet criteria for species recognition. They are phenotypically distinct [25], with behavioral evidence that some southern populations are cooperative breeders [47], whereas other Western Scrub-Jays breed in territorial pairs. This study demonstrates that these southern populations are reciprocally monophyletic to other interior populations in mtDNA and well-differentiated in nuclear markers.

Finally, we recommend that the remaining interior populations (*A. woodhouseii* including subspecies *texana*, *woodhouseii*, *nevadae*, *grisea*, and *cyanotis*) be elevated to species level, given their diagnostic phenotype [25], adaptive bill morphology [28], high support for monophyly in mtDNA in previous work [23,24] and this study (0.94 PP), and differentiation in nuclear markers (this study). Within this group, we do not recommend elevating the genetically distinctive Edwards Plateau population to species level at this time because our study sampled only one population. More information is needed on potential gene flow with other interior US populations, as well as a modern multivariate analysis of their phenotypic differentiation and studies of their ecological and behavioral divergence. Likewise, we do not currently recommend splitting Interior US from Northern Mexico populations, unless, at minimum, further sampling near the US-Mexico confirms that the break seen in microsatellite variation is not the result of a sampling artifact.

## Conclusions

We report a mismatch in cline widths between mtDNA and nuclear markers across a hybrid zone in Western Scrub-Jays. This result is consistent with predictions based on Haldane’s Rule, where there is less mtDNA introgression between lineages in female-heterogametic species. This study adds to a growing list of studies that support predictions of Haldane’s Rule using cline analysis of multiple loci of differing inheritance modes. Structure results of microsatellite variation confirm that there is only one major area of contact between coastal and interior lineages. Furthermore, Structure identified at least five clusters of individuals with strong spatial structuring: Pacific Slope, Interior US, Edwards Plateau, Northern Mexico, and Southern Mexico. Based on a variety of evidence from the phenotype, ecology, and genetics, we recommend elevating three lineages to species level: *A. californica* (Pacific Slope); *A. woodhouseii* (Interior US plus Edwards Plateau plus Northern Mexico); *A. sumichrasti* (Southern Mexico). The distinctive Edwards Plateau population in Texas, which was also monophyletic in mtDNA except for one individual, should be studied in greater detail given conservation concerns.

## Methods

### Specimens and DNA extraction

We obtained tissues from Western Scrub-Jays representing all known subspecies, genetic lineages, and geographic regions from existing frozen tissue specimens in museums or from new specimens (Table 2; see [30] for a complete list of the 689 specimens used in this study and associated data). We collected new specimens with United States federal permit MB101618 and Nevada state permit S3505. Field collecting complied with Occidental College’s Institutional Animal Care and Use Committee protocol R12-1011-01. We then extracted DNA from blood or tissue using a DNeasy Tissue Kit (QIAGEN Inc., Valencia, CA) according to the manufacturer’s protocols.

**Table 2:** Locality information for Western Scrub-Jays sampled for this study

**Map**	**Location**	**Coordinates**	**n**	**Subspecies**
1	Vida, Oregon, USA	44.1463, -122.5701	6	*immamis*
2	Alturas, California, USA	41.55, -120.6667	23	*superciliosa*
3	Lassen Co., California, USA	40.7047, -120.7391	1	*superciliosa*
4	Douglas City, California, USA	40.5833, -123	18	*caurina*
5	Beckwourth, California, USA	39.75, -120.3667	2	*superciliosa*
6	Poole Station Rd, Calaveras Co., California, USA	38.1, -120.6333	17	*superciliosa*
7	Martinez, Contra Costa Co., California, USA	38.0128, -122.1325	1	*oocleptica*
8	El Sobrante, Contra Costa Co., California, USA	37.9755, -122.2859	1	*oocleptica*
9	Alhambra Valley Rd., Contra Costa Co., California, USA	37.9663, -122.2059	1	*oocleptica*
10	Berkeley, California, USA	37.8788, -122.2583	1	*oocleptica*
11	Carmel Valley Rd., Alameda Co., California, USA	37.874, -121.832	1	*superciliosa*
12	Moraga 1, Contra Costa Co., California, USA	37.8598, -122.1343	7	*oocleptica*
12	Moraga 2, Contra Costa Co., California, USA	37.8653, -122.1386	1	*oocleptica*
12	Moraga 3, Contra Costa Co., California, USA	37.8602, -122.1235	1	*oocleptica*
12	Orinda, Contra Costa Co., California, USA	37.8654, -122.1519	17	*oocleptica*
13	Danville, Contra Costa, Co., California, USA	37.8307, -121.9826	1	*oocleptica*
14	San Ramon, Contra Costa Co., California, USA	37.75, -122	26	*oocleptica*
15	Dublin, Alameda Co., California, USA	37.7131, -121.9247	1	*oocleptica*
16	Purisima Creek, San Mateo Co., California, USA	37.4070, -122.4059	1	*oocleptica*
17	North Fork, Madera Co., California, USA	37.2429, -119.5421	1	*superciliosa*
18	Independence, Inyo Co., California, USA	36.7667, -118.2833	6	*superciliosa*
19	Bradley, Monterey Co., California, USA	35.8690, -120.9267	21	*californica*
20	Bodfish, Kern Co., California, USA	35.6167, -118.5	20	*superciliosa*
21	Big Bear City, San Bernadino Co., California, USA	34.3167, -116.8333	5	*obscura*
22	Pioneertown, San Bernadino Co., California, USA	34.1667, -116.5333	2	*obscura*
23	Santa Ana Mountains, Orange Co., California, USA	33.7, -117.6167	16	*obscura*
24	Love Valley, San Diego Co., California, USA	33.5833, -116.7833	12	*obscura*
25	Pinyon Flat, Riverside Co., California, USA	33.5833, -116.4667	6	*obscura*
26	Vallecito Mountains, San Bernadino Co., California, USA	33.027,-116.242	10	*obscura*
27	Mountain Springs Pass, Imperial Co., California, USA	32.6667, -116.0833	1	*obscura*
28	Jacumba, San Diego Co., California, USA	32.6333, -116.2167	7	*obscura*
29	La Rumorosa, Baja California, Mexico	32.55, -116.05	8	*obscura*
30	Potrero, San Diego Co., California, USA	32.65, -116.6167	3	*obscura*
31	La Rosa de Castilla, Baja California, Mexico	32.05, -116.1333	20	*obscura*
32	San Lucas, Baja California Sur, Mexico	27.5, -112.3333	1	*cactophila*
33	Bahia Magdalena, Baja California Sur, Mexico	24.7833, -112.1	15	*cactophila*
34	La Burrera, Baja California Sur, Mexico	23.5, -110.1167	12	*hypoleuca*
35	Virginia Mountains, Storey Co., Nevada, USA	39.4977, -119.6344	4	*superciliosa x nevadae*
36	Lousetown, Storey Co., Nevada, USA	39.3854, -119.6322	1	*superciliosa x nevadae*
37	Brunswick Cyn, Pine Nut Mountains, Douglas Co., Nevada, USA	39.1458, -119.6491	4	*superciliosa x nevadae*
38	Sunrise Pass Rd. 1, Pine Nut Mountains, Douglas Co., Nevada, USA	39.066, -119.569	2	*superciliosa x nevadae*
39	Sunrise Pass Rd. 2, Pine Nut Mountains, Douglas Co., Nevada, USA	39.0654, -119.5586	2	*superciliosa x nevadae*
40	Lebo Spring, Pine Nut Mountains, Douglas Co., Nevada, USA	39.0557, -119.5703	22	*superciliosa x nevadae*
41	Sunrise Pass Rd. 3, Pine Nut Mountains, Douglas Co., Nevada, USA	39.0523, -119.6028	2	*superciliosa x nevadae*
42	Hot Springs Mountain, Douglas Co., Nevada, USA	39.0333, -119.6333	6	*superciliosa x nevadae*
43	Pine Nut Creek, Douglas Co., Nevada, USA	38.9414, -119.7069	6	*superciliosa x nevadae*
44	Gardnerville, Douglas Co., Nevada, USA	38.8333, -119.6167	5	*superciliosa x nevadae*
45	Woodford, Alpine Co., California, USA	38.7833, -119.75	7	*superciliosa x nevadae*
46	Nye Canyon, Pine Grove Hills, Lyon Co., Nevada, USA	38.5703, -119.2083	10	*nevadae*
47	Pine Grove Hills, Lyon Co., Nevada, USA	38.5133, -119.2040	1	*nevadae*
48	Lucky Boy Pass Rd, Wassuk Range, Mineral Co., Nevada, USA	38.4549, -118.6701	1	*nevadae*
49	North Canyon, Wassuk Range, Mineral Co., Nevada, USA	38.4625, -118.6475	3	*nevadae*
50	Excelsior Mountains, Mineral Co., Nevada, USA	38.2792, -118.4844	1	*nevadae*
51	White Mountains, Inyo Co., California, USA	37.2833, -118.1667	16	*nevadae*
52	Mt Charleston, Clark Co., Nevada, USA	36.3667, -115.6333	27	*nevadae*
53	Toiyabe Mountains, Lander Co., Nevada, USA	39.3333, -117.1333	15	*nevadae*
54	Baker, Millard Co., Utah, USA	39.05, -114.0833	1	*nevadae*
55	Hualapi Mountains, Mohave Co., Arizona, USA	35.15, -113.9	2	*nevadae*
56	Drake, Yavapai Co., Arizona, USA	35, -112.25	26	*nevadae*
57	Stansbury Mountains, Tooele Co., Utah, USA	40.35, -112.5333	15	*woodhouseii*
58	Uinta Mountains, Duschesne Co., Utah, USA	40.5816, -110.0105	1	*woodhouseii*
59	Duschesne Co., Utah, USA	39.9813, -110.2620	1	*woodhouseii*
60	Bonanza, Uitah Co., Utah, USA	39.7811, -109.0260	1	*woodhouseii*
61	Whitewater, Mesa Co., Colorado, USA	38.9199, -108.4834	4	*woodhouseii*
62	Monument, Teller Co., Colorado, USA	39.0938, -104.8296	1	*woodhouseii*
63	Gardner, Huerfano Co., Colorado, USA	37.8833, -105.2	13	*woodhouseii*
64	Manzano, Valencia Co., New Mexico, USA	34.6667, -106.4667	26	*woodhouseii*
65	Fort Davis, Jeff Davis Co., Texas, USA	30.7, -104.1333	22	*woodhouseii*
66	Carta Valley, Edwards Co., Texas, USA	29.8333, -100.6833	28	*texana*
67	Villa Ocampo, Durango, Mexico	26.4667, -105.4833	12	*grisea*
68	Sombrerete, Zacatecas, Mexico	23.7, -103.75	9	*grisea*
69	Rancho Santa Rita, Jalisco, Mexico	21.45, -101.9167	19	*grisea*
70	El Diamante Pass, Coahuila, Mexico	25.3667, -100.8667	21	*cyanotis*
71	Bledos, San Luis Potosí, Mexico	21.8667, -101.15	20	*cyanotis*
72	Taxco, Guerrero, Mexico	18.5833, -99.6333	3	*remota*
73	Xocomanantlán, Guerrero, Mexico	17.55, -99.65	12	*remota*
74	San Lorenzo de Abarrados, Oaxaca, Mexico	17, -96.1667	24	*sumichrasti*

Map numbers correspond to sampling sites in figures.

### Assigning coastal vs. interior mtDNA haplotypes

Phylogenies based on mtDNA have been characterized in previous studies of Western Scrub-Jays using a limited numbers of individuals [23,24]. Our goal was to conduct deep mtDNA sampling across the range of the species and assign coastal and interior types to determine the extent of mixing within populations, and to determine whether rare long-distance dispersal events have occurred.

Instead of direct sequencing, we screened individuals for coastal versus interior mtDNA type with a rapid, diagnostic assay. We used previously sequenced individuals [24] to identify a rare-cutting restriction enzyme that differed between coastal individuals, which had the cut site, and interior individuals, which did not. Then, for new samples of unknown haplotype, we amplified the *cyt b* gene using previously published primers and PCR conditions [24]. Once completed, we added 0.5 μL of BSR-DI (2000 U/mL, New England Biolabs, Ipswitch, MA) to each reaction and incubated for one hour at 65°C, followed by visualization on an agarose gel. We scored individuals having two bands as coastal and individuals having one band as interior, employing both positive and negative controls. We ran samples where mtDNA type conflicted with geographic (sampling) location a second time to confirm the result.

### Nuclear microsatellite loci

We used microsatellites to gain insight into differentiation and gene flow of the nuclear genome and to score individuals as potential hybrids (given that mtDNA is haploid and provides no information as to the hybrid ancestry of individuals). Previously characterized microsatellite loci have been isolated from the Florida Scrub-Jay [48]. From these loci, we chose a panel of 14 loci that amplified well and were variable among Western Scrub-Jays. To reduce processing time and cost while maintaining data quality, we pooled up to three loci and amplified them together (multiplexing) with a single dye [49], after first confirming that allele size classes for pooled loci did not overlap.

After amplification, we determined fragment size using an ABI PRISM 3730 capillary sequencer and analyzed the resulting data using Geneious v. 6.0.4 (Biomatters). We called alleles objectively by creating bins based on known microsatellite repeat motifs [48], and assessed artifacts after genotyping (e.g., stuttering, null alleles, big-allele drop-out) using Microchecker [50]. To ensure that microsatellite loci were evolving under neutral processes, we assessed each locus for deviation from Hardy-Weinberg equilibrium (HWE) using GenePop on the Web (http://genepop.curtin.edu.au). Strong population structure can lead to false positives for deviations from HWE when populations are pooled (Wahlund Effect), so we assessed HWE in each population at each locus individually, applying Bonferroni correction to control for false positives resulting from the effect of conducting multiple simultaneous tests. We also performed a test of linkage disequilibrium to confirm independent sorting of loci.

### Analysis of microsatellite variation for genetic clustering across the geographic range

We assessed genetic structure of microsatellite data using Structure v. 2.3.4 [51], which uses Bayesian analysis to infer the number of populations (*K*) from a group of individuals based only on their genetic variation, without prior information on where the individuals originated. We did not use the method of Evanno et al. [52] to detect the ‘true *K*’ because this method is known to underestimate genetic structure in all but cases of very strong genetic differentiation [53]. Initial tests of the Evanno et al. [52] method suggested *K* = 2 as the best model for our data; however, analysis at higher *K* revealed strong structure that was highly justified based on biology and geography. Thus, because one of our goals was discovery of the basic units of spatially structured genetic diversity, we instead used Structure to analyze successively smaller clusters of individuals at *K* = 2 until Structure could no longer find any clustering within groups, as demonstrated previously [54]. For example, when two clear geographic population clusters with strong biological justification emerged in the complete data set, we ran each of these clusters in its own analysis until *K* = 2 revealed no remaining geographical clusters. After the first run, we excluded hybrid populations (identified as populations with both coastal and interior mtDNA types) from the analysis. We ran Structure with an admixture model, correlated allele frequencies, and a burn-in period set to 100,000 generations, followed by 500,000 generations, which was sufficient for each run to reach stationarity.

### Analysis of microsatellite variation across the hybrid zone

To assess population assignment and potential hybrid ancestry of individuals across the hybrid zone, we delimited 13 populations along a transect running northwest to southeast from the Sierra Nevada in northeastern California into west-central Nevada (Figure 2). This line conforms to prior descriptions of the contact zone [25,55,56] and our own field observations. To obtain estimates of hybrid ancestry (i.e., Q-scores) within the hybrid zone, individuals from these 13 populations were analyzed in Structure separately from all other individuals in the study using *K* = 2; their resulting Q-scores were recorded for later use in cline analysis. The Q-score values provide an overall estimate of nuclear variation that is expected to suffer less from random processes affecting individual loci. Moreover, cline analysis requires binary data or frequency values varying from 0 to 1. Due to large number of alleles at a given locus, individual microsatellite loci do not usually conform to this standard.

### Testing for phylogenetic structure of microsatellite groups using mtDNA

Previous studies that generated mtDNA phylogenies of Western Scrub-Jays did not sample deeply within populations [23,24]. Thus, to determine if nuclear DNA groups discovered in this study were discernible using mtDNA, we sequenced multiple individuals from all major groups suggested by our microsatellite results for the mtDNA *cyt b* gene, using previous protocols [24]. We combined these new data with existing data for *Aphelocoma* and close outgroups (*Calocitta formosa*, *Gymnorhinus cyanocephalus*, and *Cyanocitta stelleri*) from GenBank, and we generated a phylogeny from these sequences using BEAST v. 1.7.5 [57]. We ran the analysis using the best-fit substitution model for the *cyt b* gene determined through Akaike Information Criterion (AIC), which was HKY + I + G with six gamma categories and base frequencies estimated as determined by MrModeltest, v2 [58]. We used a strict clock with a random starting tree. We ran the chain to 1.0 × 10^6^ iterations, sampling every 1,000. The analysis converged quickly as determined by observing the plot of likelihood scores and ESS scores > 200 with Tracer. We discarded the first 1,000 of 10,000 trees as burn-in and produced a consensus tree from the remaining posterior trees using Geneious [59]. The maximum clade credibility produced with TreeAnnotator was almost identical, with key nodes mentioned in the Results having the same PP values.

### Fitting geographic clines to genetic data across the hybrid zone

We used HZAR v2.5 [60], a statistical package implemented in R, to determine the width of geographic clines estimated from mtDNA and nuclear markers, and the extent of mismatch, if any. We measured the distance between points from the coastal northwest end of the hybrid zone (site 2 in Figure 1), through the contact zone, continuing to pure interior populations to the southeast (site 52 in Figure 1). These were then compressed to a single line using HZAR. This transect likely captures the major axis of gene flow across the hybrid zone [25], with minor caveats covered in the Discussion. We estimated clines for mtDNA haplotype frequency and average Q-score per site as determined in our Structure analyses. We used these cline analyses to estimate changes of the molecular characters in local mean frequencies. We modeled the cline shape using three equations [61,62] describing a sigmoid shape at the center of the transition with two exponential decay curves on either side of the transition. We estimated several parameters, including width (*w*), center (*c*), delta (*d*, distance between the center and the tail), and tau (*t*, slope of the tail). We also incorporated the possibility that *Pmax* and *Pmin* (the “top” and “bottom” of the cline) were either fixed or free to vary. We fit three sets of five cline models using the Metropolis-Hasting algorithm in R. Model set 1 has no scaling (*Pmin* = 0, *Pmax* =1), model set 2 has fixed scaling (*Pmin* = observed minimum, *Pmax* = observed maximum), and model set 3 allows *Pmin* and *Pmax* to vary. Within each model set, scaling and tails are fixed or free to vary. We compared these models to a null model of no clinal transition.

For the two genetic datasets, we set up a covariance matrix by running each model for 1.0 × 10^6^ generations. We ran three independent chains for 9.0 × 10^6^ generations and assessed for convergence and stability by visualizing the MCMC traces. We performed model selection using corrected AIC (AICc). We estimated two log-likelihood confidence intervals around each parameter estimate. If the parameter estimate from one dataset did not overlap with the confidence interval of the parameter estimate from the other dataset, we considered them to be significantly different. We also used estimates of cline width to determine the time since contact under a neutral diffusion model solving for *t* in the equation *w* = 2.51σ√*t*[63], where *t* is the time since contact and σ is the root mean square dispersal distance from estimates in [31]. Because females and males have different dispersal distances, we used the female dispersal distance (1.5 – 5 km) to estimate time since contact using mtDNA, and we used the average dispersal estimate of males and females (0.5 – 3.0) to estimate time since contact for the nuclear dataset.

### Correlating phenotype with hybrid ancestry

We collected phenotypic data on a subset of 124 coastal and interior Western Scrub-Jay specimens within and outside the hybrid zone. Unfortunately we could not perform a cline analysis along the transect because we did not have sufficient sample sizes from pure populations at both ends of the transect. However, to determine the extent to which phenotype is correlated with nuclear DNA variation, we conducted regression analysis of phenotypic traits (plumage and morphology) with Q-scores at several spatial scales. The set of 124 specimens includes the full ranges of coastal and interior phenotypes, many hybrids, and pure individuals of each lineage with no evidence for hybrid genetic ancestry (Q-score at or close to 1 or 0). Because environment can influence phenotype when considering allopatric lineages, a more robust test of the genotype-phenotype link occurs within the hybrid zone where individuals share the same environment and there is more continuous variation in Q-scores. Here, we looked specifically at the 68 specimens that are part of the hybrid transect, as well as a more restrictive sample of 50 individuals within the core hybrid zone defined as sites 35–45 in Figure 1.

To ensure consistent measurements, one author (TCW) measured wing, tail, tarsus, bill length, bill depth, and bill width to the nearest 0.1 mm with digital calipers on all 124 birds after first verifying high repeatability scores for all traits. On a subset of 66 of these birds, another author (JEM) assessed three qualitative plumage traits known to vary between coastal and interior lineages: the amount of blue collar (reduced in interior birds); size of the eyestripe (reduced in interior birds); and color of the undertail coverts (blue-tinged in interior birds as opposed to white in coastal birds). We determined variation in plumage traits by lining up all 66 specimens at the same time and arranging them in order of trait appearance (e.g., very white undertail coverts to very blue undertail coverts). We then divided the arranged specimens into six even categories and assigned a value of 1 to 6. We repeated these steps for each trait. We analyzed morphological data with principal components analysis on the correlation matrix in Stata 10 to identify axes defining the most variation in the data. We assessed relationships between Q-scores and univariate and multivariate phenotypic traits with linear regression at each of the three spatial extents described above (full range, hybrid transect, core hybrid zone) using Stata.

## Availability of supporting data

New DNA sequences have been deposited in Genbank under accession numbers KJ835799-KJ835861. Museum catalog numbers, localities, raw microsatellite data, phenotypic data, and Q-scores for each individual are available through Dryad http://dx.doi.org/10.5061/dryad.57f48.

## Competing interests

The authors declare that they have no competing interests.

## Authors’ contributions

FCG collected samples and genetic data, conducted Structure analyses, and helped write the manuscript; JMM helped design the study, conducted cline analyses, and helped interpret results and write the manuscript; CC helped design the study, provided samples, and helped revise the manuscript; ATP helped design the study, provided samples, and helped revise the manuscript; BCF contributed to data acquisition and helped revise the manuscript; TCW helped collect genetic data, collected and analyzed phenotypic data, and helped revise the manuscript; JEM designed the study, collected samples and genetic and phenotypic data, analyzed genetic and phenotypic data, and helped write the manuscript. All authors read and approved the final manuscript.

## Authors’ information

FCG conducted part of this research for her Master’s thesis at the Moore Laboratory of Zoology at Occidental College; JMM is the Collections Manager at the Moore Laboratory of Zoology; CC is Staff Curator of Birds at the Museum of Vertebrate Zoology at Berkeley; ATP is Distinguished Professor in the Ecology and Evolutionary Biology Department and Curator of Ornithology at the Biodiversity Institute at Kansas University; BCF is an Assistant Research Scientist at UCLA; TCW was an undergraduate at Louisiana State University while conducting this research; JEM is Curator of Birds and Mammals and Director of the Moore Laboratory of Zoology and an Assistant Professor in the Biology Department at Occidental College.

## Supplementary Material

Additional file 1 Loadings for principal components analysis. Click here for file
