# Supplementary material for: Speciation in Western Scrub-Jays, Haldane’s rule, and genetic clines in secondary contact
Source: BMC Evol Biol. 2014 Jun 17;14:135. doi: 10.1186/1471-2148-14-135 (PMC4078322; doi:10.1186/1471-2148-14-135)
Supplement: Additional file 1 — Loadings for principal components analysis. [file 1471-2148-14-135-S1.docx]

Additional File 1. Loading plot for principal components analysis of morphological data.
